# Effects of physical activity during pregnancy on preterm delivery and mode of delivery: The Japan Environment and Children’s Study, birth cohort study

**DOI:** 10.1371/journal.pone.0206160

**Published:** 2018-10-29

**Authors:** Mio Takami, Akiko Tsuchida, Ayako Takamori, Shigeru Aoki, Mika Ito, Mika Kigawa, Chihiro Kawakami, Fumiki Hirahara, Kei Hamazaki, Hidekuni Inadera, Shuichi Ito, and the Japan Environment & Children’s Study (JECS) Group

**Affiliations:** 1 Perinatal Center for Maternity and Neonate, Yokohama City University Medical Center, Japan; 2 Kanagawa Regional Center for JECS, Graduate School of Medicine, Yokohama City University, Kanagawa, Japan; 3 Department of Public Health, Faculty of Medicine, University of Toyama, Toyama, Japan; 4 Toyama Regional Center for JECS, University of Toyama, Toyama, Japan; 5 Clinical Research Center, Saga University Hospital, Saga, Japan; 6 Department of Obstetrics and Gynecology, Faculty of Medicine, University of Toyama, Toyama, Japan; 7 Faculty of Health and Welfare, Kanagawa University of Human Services, Kanagawa, Japan; 8 Department of Obstetrics and Gynecology, Yokohama City University Hospital, Kanagawa, Japan; 9 Department of Pediatrics, Graduate School of Medicine, Yokohama City University, Kanagawa, Japan; University of Missouri Columbia, UNITED STATES

## Abstract

**Background:**

The aim of this study was to examine how physical activity (PA) before and during pregnancy influences pregnancy outcomes, particularly preterm delivery and mode of delivery.

**Methods:**

This study was based on the Japan Environment and Children’s Study. A total of 92,796 pregnant women who gave birth to live singleton babies were included. Information on mean PA per week during pregnancy was extracted from the responses to questionnaires completed by women during the second and third trimesters of pregnancy. Information on PA before pregnancy was obtained from questionnaires answered based on recall at participation. The level of PA was stratified into the following quartiles for categorical analysis: Very low, Low, Medium, and High. Pregnancy outcomes, gestational age at delivery (whether preterm delivery or not), and mode of delivery (spontaneous, instrumental, or caesarean delivery) were compared between the different groups adjusted for multiple covariates.

**Results:**

With respect to PA during pregnancy, the risk of preterm delivery and instrumental delivery increased significantly in the Very low group compared to that in the Medium group (odds ratios [OR] 1.16, 95% confidence interval [CI], 1.05–1.29; OR 1.12, 95% CI, 1.03–1.22, respectively). Moreover, the risks of caesarean delivery in the Low group and instrumental delivery in the High group were significantly higher than the risks in the Medium group (OR 1.07, 95% CI, 1.00–1.15; OR 1.12, 95% CI, 1.02–1.22, respectively). In contrast, with respect to PA before pregnancy, there were no statistically significant differences when the other groups were compared to the Medium group.

**Conclusions:**

Pre-pregnancy PA has no negative effects on preterm birth and caesarean delivery. In contrast, both may be affected by PA during pregnancy because a low level of PA appears to slightly increase the risk of preterm delivery and operative delivery (caesarean and instrumental).

## Introduction

Physical activity during pregnancy contributes to maintaining and improving fitness, and is associated with cardiorespiratory fitness [1–3], prevention of low back pain and urinary incontinence [4–6], reduced symptoms of depression [7], and control of gestational weight gain [8]. In the absence of complications that constitute contraindications for physical activity, pregnant women should be encouraged to engage in a range of recreational activities, which appear to be beneficial and safe for both the mother and fetus [1–3]. It is clear that physical activity provides benefits for maternal health and quality of life (QOL). The American Congress of Obstetricians and Gynecologists (ACOG) recommends that all pregnant women should be engaged in moderate-intensity exercise (such as aerobics) for 30 minutes or more per day on most, if not all days of the week [1–3].

However, it is difficult to accurately assess free-living physical activity. Moreover, reports of studies on the influence of physical exercise on pregnancy outcomes usually involve a small number of subjects [1,9,10]. In addition, discussions on the impact of physical activity on the mother and fetus are inadequate and not universally accepted. Furthermore, few studies have investigated the influence of pre-pregnancy physical activity on pregnancy outcomes [11].

The aim of the present study was to examine how physical activity and exercise habits before and during pregnancy influence pregnancy outcome, particularly preterm delivery and mode of delivery in a large nationwide population based study of approximately 100,000 Japanese pregnant women.

## Materials and methods

This study was performed based on the “Japan Environment and Children’s Study (JECS)” led by the Japanese Ministry of the Environment. The JECS is an ongoing nation-wide birth cohort study on deliveries for which approximately 100,000 pregnant women have been recruited. The main objective of the JECS is to determine the effect of environmental factors on children’s health and development in the fetal period and after delivery. The JECS protocol was approved by the Institutional Review Board on epidemiological studies of the Ministry of the Environment, and by Ethics Committees of all participating institutions.

In the JECS, apart from obtaining data on the wide variety of environmental hazards which children face, detailed information on lifestyle factors of pregnant women and data on pregnancy outcomes can also be obtained. Using such valuable data, we have investigated the impact of physical activity on pregnancy outcomes in this study. More details of the recruitment and collecting data protocols of JECS have been published elsewhere [12,13].

### Study population and inclusion criteria

From January 2011 to March 2014, pregnant women living in the 15 study regions were recruited to cover wide geographical areas in Japan. Data of 104,102 fetuses and their mothers were recorded. The present study is based on the data set of jecs-ag-20160426, which was released in June 2016. An explanation of the study was given to each participant from whom written informed consent was obtained. The JECS was conducted in accordance with the Helsinki Declaration and other nationally valid regulations and guidelines. The questionnaire was completed by women in the first trimester and in the second and third trimesters of pregnancy. Information on physical activity habits and confounding factors was extracted from the responses to the questionnaires. In addition, we obtained data on pregnancy outcomes from the medical records.

**Fig 1:**
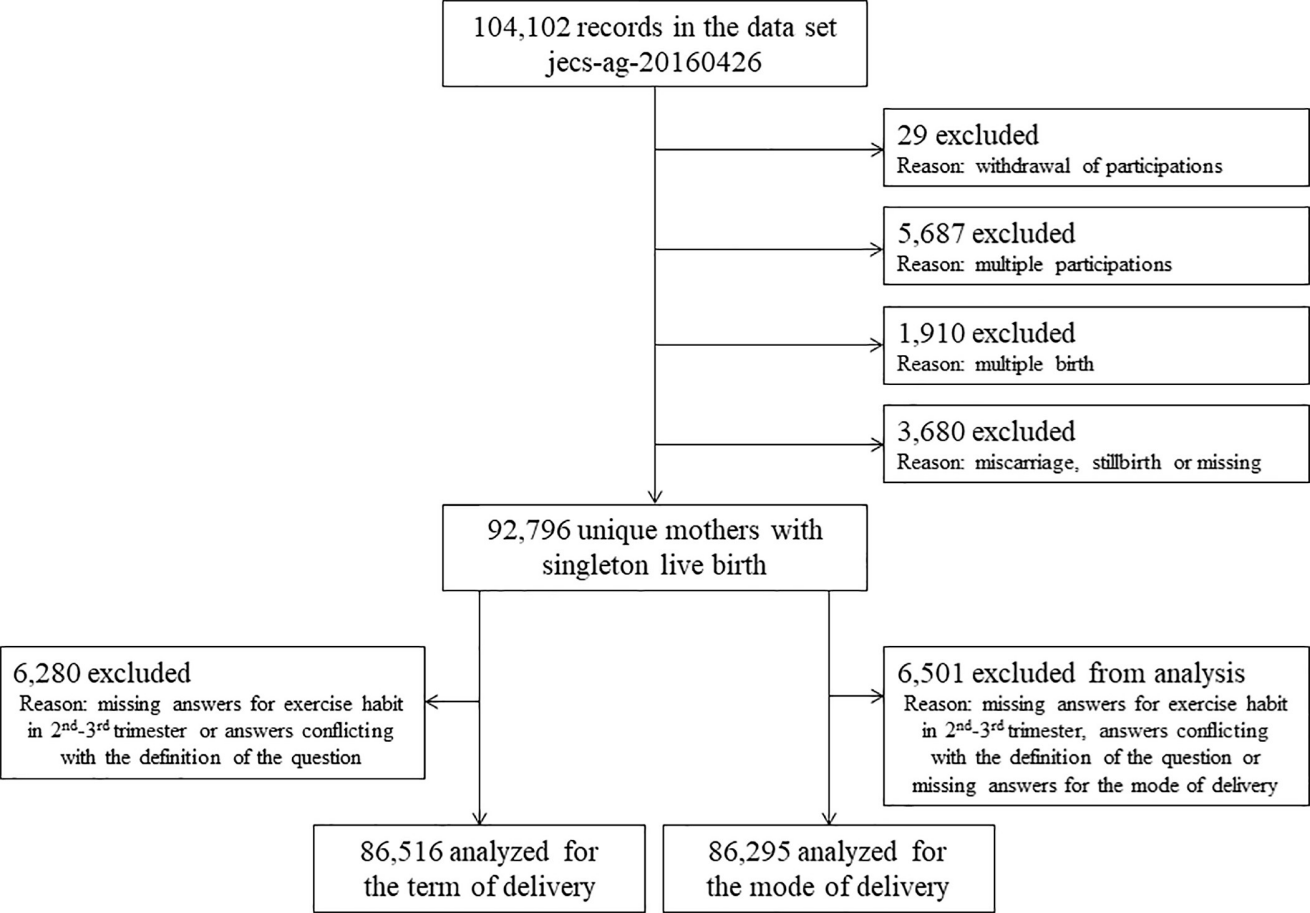
Participants inclusion flowchart with regard to the analysis of physical activities during pregnancy. The study included 92,796 pregnant women from the dates of Japan Environment and Children’s Study (JECS).

Twenty-nine women dropped out of the study, 5,687 women with multiple participations were registered from the second assessment onwards, 1,910 women with multiple pregnancies, and 3,680 women with miscarriages or still births were excluded; hence, a total of 92,796 pregnant women with singleton live birth were included in the study (Fig 1). This study was approved by the ethics committee of Yokohama City University, Yokohama City University Medical Center, University of Toyama, and the JECS Programme Office.

### Questionnaires about physical activity

We used the Japanese version (the usual week, short, self-administered version) of the international physical activity questionnaire (IPAQ) to query subjects about physical activity during pregnancy. Then, using the method of Murase et al. [14], we calculated physical activity in terms of MET h/week (metabolic equivalent of a task measured as the number of hours per week) [14–16]. Physical activity as defined in the IPAQ encompasses all time spent being physically active including work-related activities, doing housework, and leisure-time activities. Therefore, exercise such as swimming, running, walking is included in physical activity.

In the JECS, pregnant women answered questionnaires about their “mean physical activity per week before pregnancy” based on recall at the time of participating in the JECS (median: 15 weeks of gestation). They also answered questionnaires about the “mean physical activity per week during pregnancy” in the second and third trimesters (median: 27 weeks of gestation). In this study, responses to questions on “physical activity per week during pregnancy” were used in the analysis. Similarly, responses to questions on “physical activity per week before pregnancy” were also analyzed. The level of physical activity was stratified into the following quartiles for categorical analysis: Very low, Low, Medium, and High.

### Main outcomes

Pregnancy outcome was assessed taking into consideration the gestational age at delivery (whether or not a preterm delivery) and mode of delivery (spontaneous, instrumental, or Caesarean). Preterm delivery was defined as delivery at or after 22 weeks and at less than 37 weeks of gestation. Instrumental delivery was defined as vacuum delivery or forceps delivery. The level of physical activity was categorized into the 4 groups mentioned above and pregnancy outcomes were compared between the different groups.

### Statistical analysis

The following variables were considered in the analysis: maternal age at delivery, marital status, education, household income, drinking status in the second and third trimesters, smoking status in the second and third trimesters, passive smoking in the second and third trimesters, total calorie intake, folic acid intake; pregnancy-associated abnormalities, namely, threatened abortion, threatened preterm delivery, gestational diabetes, gestational hypertension, intrauterine infection; and maternal complications, namely, hypertension, diabetes, fibroids, cervical cancer, pre-pregnancy body mass index (BMI), weight gain during pregnancy, gestational age at delivery, and scores for measuring health concept during pregnancy, specifically the QOL summary score (physical component summary [PCS]) [17].

The maternal age at delivery was categorized into the following age groups <20, 20–24, 25–29, 30–34, 35–39, or ≥40 years. In the model used to examine the variables for primipara or multipara and preterm delivery outcome, variables were stratified into categories incorporating the status of history of preterm delivery: “primipara”, “multipara with no history of preterm delivery”, and “multipara with history of preterm delivery”. Similarly, variables were stratified into categories incorporating the status of history of Caesarean delivery: “primipara”, “multipara with no history of Caesarean delivery”, and “multipara with a history of Caesarean delivery”.

The Pearson’s chi square test was used to test categorical variables, and one-way analysis of variance was used for continuous variables. Multivariate logistic regression analysis was adjusted for the variables and the odds ratios (OR) were calculated. The contribution ratio of the variables was estimated by the likelihood ratio test. All the statistical analyses were performed using SAS version 9.4 software (SAS Institute Inc., Cary, NC), and p<0.05 was considered statistically significant.

## Results

Of a total of 92,796 pregnant women included in the study, those with missing documentation on gestational age at delivery or mode of delivery were excluded. Moreover, those with missing responses to the questionnaire regarding exercise habits during pregnancy and those with answers conflicting with the definition of the question (for example, responses of “at least 8 days” when asked the number of days a week of exercising, or responses of “at least 24 hours” when asked the number of hours a day of exercising) were excluded. Finally, preterm delivery was investigated in 86,516 pregnant women, and mode of delivery in 86,295 (Fig 1). The median level of physical activity per week (METs-hr/week) during pregnancy was 8.2 METs-hr/week (range 0–949.2) (Table 1).

**Table 1:** Amounts of physical activity during pregnancy (Mets・h/w).

	Mets・h/w
	n = 86,516
Maximum	949.2
Upper quartile	23.1
Median	8.2
Lower quartile	1.1
Minimum	0

The level of physical activity was divided into the following 4 groups: Very low (0–1.1 METs-hr/week), Low (1.1–8.2 METs-hr/week), Medium (8.2–23.1 METs-hr/week), and High (≥23.1 METs-hr/week).

The frequency of preterm delivery in the total deliveries in this study was 4.5%. Moreover, the frequencies of spontaneous delivery, Caesarean delivery, and instrumental delivery were 75.2%, 18.8%, and 6.0%, respectively. Details of the maternal characteristics, including the variables examined, are presented in Table 2.

**Table 2:** Maternal characteristics according to the term of delivery (n = 86,516) or the mode of delivery (n = 86,295).

	Term of delivery				Mode of delivery					
	Full term n = 82,592 (95.5%)		Preterm n = 3,924 (4.5%)		Spontaneous delivery n = 64,930 (75.2%)		Caesarean delivery n = 16,198 (18.8%)		Instrumental delivery n = 5,167(6.0%)	
	n	(%)	n	(%)	n	(%)	n	(%)	n	(%)
**Physical activity level during pregnancy**										
Very Low	20,875	(25.3)	1,277	(32.5)	16,306	(25.1)	4,443	(27.4)	1,357	(26.3)
Low	19,607	(23.7)	839	(21.4)	15,318	(23.6)	3,876	(23.9)	1,212	(23.5)
Medium	21,426	(25.9)	898	(22.9)	16,927	(26.1)	4,004	(24.7)	1,315	(25.5)
High	20,684	(25.0)	910	(23.2)	16,379	(25.2)	3,875	(23.9)	1,283	(24.8)
**Age at delivery**										
<20	717	(0.9)	26	(0.7)	610	(0.9)	89	(0.6)	43	(0.8)
20–25	7,406	(9.0)	284	(7.2)	6,139	(9.5)	987	(6.1)	547	(10.6)
25–29	22,870	(27.7)	922	(23.5)	18,798	(29.0)	3,468	(21.4)	1,471	(28.5)
30–34	29,334	(35.5)	1,336	(34.1)	23,180	(35.7)	5,575	(34.4)	1,825	(35.3)
35–40	18,538	(22.5)	1,109	(28.3)	13,749	(21.2)	4,783	(29.5)	1,068	(20.7)
> = 40	3,724	(4.5)	247	(6.3)	2,453	(3.8)	1,295	(8.0)	212	(4.1)
Mean (SD)	31.2	(5.0)	32.0	(5.1)	30.9	(5.0)	32.5	(5.1)	30.8	(5.1)
**Current marital status**										
Married	78,170	(95.5)	3,702	(95.5)	61,440	(95.6)	15,382	(95.7)	4,838	(94.2)
Single (Never married)	2,984	(3.7)	120	(3.1)	2,324	(3.6)	518	(3.2)	258	(5.0)
Divorced or Widowed	689	(0.8)	54	(1.4)	532	(0.8)	170	(1.1)	38	(0.7)
**Education(year)**										
<12	29,646	(36.0)	1,460	(37.4)	23,304	(36.0)	5,900	(36.6)	1,841	(35.8)
12–14	34,579	(42.0)	1,638	(42.0)	26,999	(41.7)	6,928	(43.0)	2,193	(42.6)
>14	18,064	(22.0)	805	(20.6)	14,392	(22.3)	3,302	(20.5)	1,112	(21.6)
**Annual household income(yen)**										
<4 million	30,717	(39.9)	1,499	(41.0)	24,264	(40.1)	5,999	(39.6)	1,877	(39.3)
4–6 million	25,464	(33.1)	1,208	(33.1)	20,118	(33.3)	4,927	(32.6)	1,565	(32.8)
>6 million	20,780	(27.0)	947	(25.9)	16,117	(26.6)	4,207	(27.8)	1,330	(27.9)
**Alcohol intake during 2nd 3rd trimester**										
Never drinker	27,198	(33.2)	1,389	(35.8)	21,511	(33.4)	5,308	(33.0)	1,704	(33.1)
Quit Before Pregnancy	13,570	(16.6)	639	(16.5)	10,588	(16.4)	2,783	(17.3)	791	(15.4)
Quit After Pregnancy	38,890	(47.5)	1,772	(45.6)	30,456	(47.3)	7,556	(47.0)	2,550	(49.6)
Current drinker	2,292	(2.8)	85	(2.2)	1,848	(2.9)	426	(2.7)	97	(1.9)
**Smoking status during 2nd 3rd trimester**										
Never Smoker	47,537	(58.0)	2,181	(56.2)	37,426	(58.1)	9,055	(56.3)	3,113	(60.6)
Quit Before Pregnancy	19,312	(23.6)	941	(24.2)	15,192	(23.6)	3,938	(24.5)	1,065	(20.7)
Quit After Pregnancy	11,473	(14.0)	535	(13.8)	8,915	(13.8)	2,305	(14.3)	759	(14.8)
Current Smoker	3,645	(4.5)	225	(5.8)	2,883	(4.5)	781	(4.9)	197	(3.8)
**Exposure to tobbaco smoke during 2nd 3rd trimester**										
No	51,226	(62.1)	2,395	(61.2)	40,324	(62.2)	9,969	(61.7)	3,184	(61.7)
1/week	9,938	(12.1)	411	(10.5)	7,773	(12.0)	1,910	(11.8)	637	(12.3)
2-3/week	6,799	(8.3)	315	(8.1)	5,357	(8.3)	1,286	(8.0)	448	(8.7)
4-6/week	4,054	(4.9)	185	(4.7)	3,119	(4.8)	842	(5.2)	270	(5.2)
Everyday	10,428	(12.7)	605	(15.5)	8,234	(12.7)	2,158	(13.4)	624	(12.1)
**Threatened abortion**										
No	72,892	(88.3)	3,337	(85.0)	57,449	(88.5)	14,143	(87.3)	4,429	(85.7)
Yes	9,700	(11.7)	587	(15.0)	7,481	(11.5)	2,055	(12.7)	738	(14.3)
**Threatened premature delivery**										
No	67,781	(82.1)	2,087	(53.2)	52,900	(81.5)	12,611	(77.9)	4,154	(80.4)
Yes	14,811	(17.9)	1,837	(46.8)	12,030	(18.5)	3,587	(22.1)	1,013	(19.6)
**Gestational diabetes**										
No	80,423	(97.4)	3,764	(95.9)	63,385	(97.6)	15,526	(95.9)	5,059	(97.9)
Yes	2,169	(2.6)	160	(4.1)	1,545	(2.4)	672	(4.2)	108	(2.1)
**Gestational hypertension**										
No	80,380	(97.3)	3,410	(86.9)	63,498	(97.8)	15,083	(93.1)	4,995	(96.7)
Yes	2,212	(2.7)	514	(13.1)	1,432	(2.2)	1,115	(6.9)	172	(3.3)
**Intrauterine infection**										
No	82,149	(99.5)	3,855	(98.2)	64,643	(99.6)	16,004	(98.8)	5,137	(99.4)
Yes	443	(0.5)	69	(1.8)	287	(0.4)	194	(1.2)	30	(0.6)
**Hypertension**										
No	81,742	(99.0)	3,691	(94.1)	64,384	(99.2)	15,713	(97.0)	5,119	(99.1)
Yes	850	(1.0)	233	(5.9)	546	(0.8)	485	(3.0)	48	(0.9)
**Diabetes**										
No	81,756	(99.0)	3,837	(97.8)	64,371	(99.1)	15,886	(98.1)	5,118	(99.1)
Yes	836	(1.0)	87	(2.2)	559	(0.9)	312	(1.9)	49	(1.0)
**Uterine fibroids**										
No	77,248	(94.0)	3,559	(91.3)	61,517	(95.2)	14,240	(88.3)	4,842	(94.0)
Yes	4,952	(6.0)	341	(8.7)	3,075	(4.8)	1,894	(11.7)	312	(6.1)
**Cervical cancer**										
No	81,606	(99.3)	3,798	(97.4)	64,100	(99.2)	15,965	(99.0)	5,120	(99.3)
Yes	594	(0.7)	102	(2.6)	492	(0.8)	169	(1.1)	34	(0.7)
**Previous preterm delivery**										
Primipara	34,440	(42.8)	1,576	(41.2)	25,621	(40.4)	6,725	(42.6)	3,562	(71.1)
Multipara-no	43,895	(54.5)	1,779	(46.5)	36,067	(56.9)	8,142	(51.6)	1,371	(27.4)
Multipara-yes	2,206	(2.7)	473	(12.4)	1,674	(2.6)	925	(5.9)	75	(1.5)
**Previous caesarean delivery**										
Primipara	34,440	(42.8)	1,576	(41.2)	25,621	(40.4)	6,725	(42.6)	3,562	(71.1)
Multipara-no	39,316	(48.8)	1,701	(44.4)	37,346	(58.9)	2,220	(14.1)	1,364	(27.2)
Multipara-yes	6,785	(8.4)	551	(14.4)	395	(0.6)	6,847	(43.4)	82	(1.6)
	Mean	(SD)	Mean	(SD)	Mean	(SD)	Mean	(SD)	Mean	(SD)
**BMI before regnancy**										
	21.2	(3.3)	21.5	(3.8)	21.0	(3.1)	22.0	(3.9)	20.9	(3.0)
**Weight gain during pregnancy (kg)**										
	10.4	(4.0)	8.0	(4.2)	10.4	(3.9)	9.9	(4.4)	10.5	(4.0)
**Gestational week at delivery(w)**										
	39.1	(1.1)	34.6	(2.4)	39.1	(1.3)	37.8	(2.0)	39.3	(1.2)
**SF-8 Physical Component Summary during pregnancy**										
	45.8	(6.1)	44.5	(7.2)	45.8	(6.2)	45.3	(6.4)	45.7	(6.1)
**Total carorie intake during pregnancy (kcal)**										
	1,735.7	(777.2)	1,733.3	(755.5)	1,736.8	(781.0)	1,750.2	(786.0)	1,677.3	(678.8)
**Folic acid intake during pregnancy (ug)**										
	258.6	(167.7)	258.4	(157.1)	258.6	(166.9)	261.0	(169.2)	250.4	(165.3)

Table 3 shows the association between physical activity during pregnancy and preterm delivery. Compared to the Medium group, no significant difference was found in the incidence of preterm delivery in the Low and High groups, however, that of the Very low group increased significantly (OR 1.16, 95% confidence interval [CI],1.05–1.29, p = 0.004). Similar results were obtained regarding preterm delivery at less than 32 weeks of gestation.

**Table 3:** Association between physical activity level during pregnancy and preterm delivery.

	Number of cases		Gestational week at delivery among cases		Crude			Adjusted		
	n	(%)	Mean	(SD)	OR	[95%CI]	p value	OR	[95%CI]	p value
**Preterm delivery vs fullterm delivery**										
Very low	1,277	(5.76)	1,277	(5.76)	**1.46**	**[1.34–1.59]**	<0.001*	**1.16**	**[1.05–1.29]**	0.004*
Low	839	(4.10)	839	(4.10)	1.02	[0.93–1.12]	0.672	0.97	[0.87–1.08]	0.617
Medium	898	(4.02)	898	(4.02)	reference			reference		
High	910	(4.21)	910	(4.21)	1.05	[0.96–1.15]	0.313	0.99	[0.89–1.10]	0.872
**Early preterm delivery**^†^**vs fullterm delivery**										
Very low	168	(0.80)	168	(0.80)	**2.05**	**[1.58–2.68]**	<0.001*	**1.38**	**[1.01–1.88]**	0.043*
Low	91	(0.46)	91	(0.46)	1.18	[0.88–1.60]	0.266	1.11	[0.79–1.56]	0.556
Medium	84	(0.39)	84	(0.39)	reference			reference		
High	82	(0.39)	82	(0.39)	1.01	[0.74–1.37]	0.943	0.88	[0.61–1.25]	0.470

*p<0.05

^†^preterm delivery at less than 32 weeks of gestation.

Adjusted for maternal age, current marital status, education, annual household income, alcohol intake during 2^nd^ 3^rd^ trimester, smoking status during 2^nd^ 3^rd^ trimester, exposure to tobbaco smoke during 2^nd^ 3^rd^ trimester, threatened abortion, threatened premature delivery, gestational diabetes, gestational hypertension, intrauterine infection, hypertension, diabetes, uterine fibroids, cervical cancer, previous preterm delivery, previous caesarean delivery, BMI before pregnancy, weight gain during pregnancy, SF-8 Physical Component Summary during pregnancy, total carorie intake during pregnancy, folic acid intake during pregnancy.

Table 4 shows the association between physical activity during pregnancy and mode of delivery. Compared to the Medium group, no significant difference was found in the incidence of Caesarean delivery in the High and Very low groups. However, that of the Low group (OR 1.07, 95%CI, 1.00–1.15, p = 0.049) increased significantly. Moreover, regarding instrumental delivery, compared to the Medium group, no significant difference was found in the incidence of instrumental delivery in the Low group. However, in the Very low (OR 1.12, 95%CI, 1.03–1.22, p = 0.011) and High (OR 1.12, 95%CI, 1.02–1.22, p = 0.014) groups, the risk increased significantly.

**Table 4:** Association between physical activity level during pregnancy and mode of delivery.

	Number of cases		Crude			Adjusted		
	n	(%)	OR	[95%CI]	p value	OR	[95%CI]	p value
**Caesarean delivery vs spontaneous delivery**								
Very low	4,443	(21.41)	**1.15**	**[1.10–1.21]**	<0.001*	1.05	[0.98–1.13]	0.146
Low	3,876	(20.19)	**1.07**	**[1.02–1.12]**	0.007*	**1.07**	**[1.00–1.15]**	0.049*
Medium	4,004	(19.13)	reference			reference		
High	3,875	(19.13)	1.00	[0.95–1.05]	0.995	1.03	[0.95–1.10]	0.490
**Instrumental delivery vs spontaneous delivery**								
Very low	1,357	(7.68)	1.07	[0.99–1.16]	0.087	**1.12**	**[1.03–1.22]**	0.011*
Low	1,212	(7.33)	1.02	[0.94–1.10]	0.658	1.04	[0.95–1.14]	0.391
Medium	1,315	(7.21)	reference			reference		
High	1,283	(7.26)	1.01	[0.93–1.09]	0.839	**1.12**	**[1.02–1.22]**	0.014*
**Vacuum delivery vs spontaneous delivery**								
Very low	1,313	(7.45)	**1.09**	**[1.01–1.18]**	0.037*	**1.13**	**[1.03–1.24]**	0.007*
Low	1,169	(7.09)	1.03	[0.95–1.12]	0.447	1.05	[0.96–1.15]	0.298
Medium	1,251	(6.88)	reference			reference		
High	1,241	(7.04)	1.03	[0.95–1.11]	0.549	**1.13**	**[1.03–1.23]**	0.008*
**Forceps delivery vs spontaneous delivery**								
Very low	44	(0.27)	0.71	[0.48–1.04]	0.083	0.90	[0.59–1.36]	0.603
Low	43	(0.28)	0.74	[0.50–1.09]	0.129	0.85	[0.55–1.30]	0.449
Medium	64	(0.38)	reference			reference		
High	42	(0.26)	**0.68**	**[0.46–1.00]**	0.049*	0.88	[0.57–1.36]	0.565

*p<0.05

Adjusted for maternal age, current marital status, education, annual household income, alcohol intake during 2^nd^ 3^rd^ trimester, smoking status during 2^nd^ 3^rd^ trimester, exposure to tobbaco smoke during 2^nd^ 3^rd^ trimester, threatened abortion, threatened premature delivery, gestational diabetes, gestational hypertension, intrauterine infection, hypertension, diabetes, uterine fibroids, cervical cancer, previous caesarean delivery, BMI before pregnancy, weight gain during pregnancy, SF-8 Physical Component Summary during pregnancy, total carorie intake during pregnancy, folic acid intake during pregnancy.

Analogously to the analysis of physical activity during pregnancy, pre-pregnancy physical activity was also investigated on preterm delivery and mode of delivery. However, compared to the Medium group, there were no statistically significant differences in any of the other groups (See S1 Fig and S1–S4 Tables online).

## Discussion

In this study, pre-pregnancy physical activity did not affect preterm birth and caesarean delivery. On the other hand, both were affected by physical activity during pregnancy. In particular, a low level of physical activity during pregnancy increased the risk of preterm birth and operative delivery (i.e., Caesarean delivery, instrumental delivery). Compared to the Medium group, the risk of preterm delivery and instrumental delivery increased significantly in the Very low group. Moreover, the risk of Caesarean delivery in the Low group, and that of instrumental delivery in the High group were significantly higher than in the Medium group.

Moderate physical activity during pregnancy was not a risk factor of preterm delivery; however, a markedly low level of physical activity during pregnancy was a risk factor for preterm delivery. In the past, physical activity was assumed to cause physical and mental stress mediated by the sympathetic nervous system, resulting in increased catecholamine and prostaglandin levels. Uterine muscle activity is stimulated, causing the uterus to contract, which is a risk of preterm delivery [18–21]. Recently, however, many reports have stated that moderate exercise does not increase the risk of preterm delivery [1,9,22–26]. In 2016, Sanabria-Martinez et al. [26] conducted a review of 14 randomized clinical trials (RCTs) assessing the influence of exercise during pregnancy on neonatal outcomes in 3044 pregnancies. There was no significant difference in gestational age at delivery and incidences of threatened preterm labor and preterm delivery were low and similar between the groups (physical activity intervention group 29/1,548 = 1.9%, control group 26/1,496 = 1.7%). In contrast, Kramer and McDonald [27] reported that although exercise during pregnancy did not negatively affect gestational age at delivery, the risk of preterm birth increased, albeit without a statistically significant difference (risk ratio [RR] 1.8, 95% CI, 0.35–9.57). The Authors mentioned that the data available was insufficient to establish the recommendation of effective exercise, and that methodologically higher quality studies involving large cohorts are needed. Studies in which threatened abortion and threatened preterm delivery have been considered as confounding factors in cases that are refrained from performing exercises are scarce. In the present high-quality statistical study involving a large cohort and multiple covariates, the risk of preterm delivery was shown to be higher in the Extremely low physical activity during pregnancy group, while this risk was not increased in the High physical activity group. This finding does not contradict that of previous research.

Low level of physical activity during pregnancy slightly increased the risk of Caesarean delivery. To date, many studies investigating the association between physical activity and mode of delivery have concluded that, although there are some reports stating that exercise does not affect the mode of delivery [28–30], physical activity during pregnancy reduces the risk of Caesarean delivery and increases the probability of vaginal delivery [10,31–35]. For example, in 2015, Poyatos et al. [10] conducted a systemic review of 10 RCTs to examine the influence of exercise during pregnancy on mode of delivery, followed by a meta-analysis involving 3,160 pregnant women from these trials. They reported that physical exercise during pregnancy increases the likelihood of spontaneous vaginal delivery (RR 1.12). In particular, exercise in the second and third trimesters increases the possibility of spontaneous delivery (RR 1.14) and lowers the risk of caesarean delivery (RR 0.66). However, these were interventional studies that did not consider activities such as leisure-time activities, housework etc., and underlined that exercise programs varied in the different studies. In the present study investigating overall mean daily physical activity, moderate or higher level of exercise did not increase the risk of caesarean delivery, rather low level of exercise increased the risk slightly. Our finding is in agreement with what has been reported to date.

The risk of instrumental delivery increased slightly in the high level of physical activity and even in the extremely low level of physical activity. To date, there has been no large-scale study evaluating the impact of physical activity on instrumental delivery and reports are also sparse [28,29,31,32]. In 2014, Domenjoz et al. [34] performed a meta-analysis on 8 RCTs that involved a total of 2,083 pregnant women in their review on the influence of physical activity during pregnancy on modes of delivery considering instrumental delivery. They concluded that physical activity during pregnancy does not influence the risk of instrumental delivery (RR 1.00). Despite an extensive literature survey, we could not find previous epidemiological studies that show the association between momentum and instrumental delivery and thus provide evidence supporting the present study, or previous studies that analogized the mechanism by which physical activity affects instrumental delivery. Compared to caesarean delivery, the indication for instrumental delivery depends much more on the judgment of the facility and the attending physician. Although the present study used more covariates than any other study to date, the indication for instrumental delivery remains unclear, as we could not take into consideration main indication factors, such as fetal distress, arrested labor, etc. Therefore, with respect to the relationship between physical activity and instrumental delivery, further consideration is needed after clarifying the indications for instrumental delivery.

An extremely low level of physical activity during pregnancy significantly increases the risk of preterm delivery and instrumental delivery. Conversely, moderate physical activity during pregnancy was shown not to increase the risk of preterm delivery or Caesarean delivery. Although these findings are well-established, the strength of our study is that we have confirmed these results in a large-scale Asian based cohort.

Physical activity levels during pregnancy are commonly lower compared to those before pregnancy and various countries report that many pregnant women are not performing the level of physical activity recommended by ACOG [36–38]. However, moderate physical activity during pregnancy is recommended as it is considered beneficial with regards to promotion of maternal health and pregnancy outcomes, as assessed by gestational age at delivery and mode of delivery. Considering that pre-pregnancy physical exercise had no impact on pregnancy outcomes, this presents an opportunity to encourage women who normally do not habitually exercise to change their exercise habits even after becoming aware that they are pregnant.

Regardless of the pregnancy status, performing moderate (3–6 METs) physical activity for 150 min per week is recommended globally to maintain health for substantial health benefits (Physical Activity Guidelines for Americans) [39,40]. Performing double this amount of physical activity, which is equivalent to 7.5–15 METs-hr/week, contributes even further to improve health. This level of physical activity is almost equivalent to the level of physical activity of the Moderate group in this study. The results of this study indicated that women who actually perform this recommended level of physical activity have a higher probability of term delivery and spontaneous vaginal delivery. Thus, moderate physical activity of approximately 7.5–15 METs-hr/week is an appropriate level of physical activity when not pregnant and even during pregnancy as it has a good impact not only on the maternal fitness but also on pregnancy outcomes.

The strengths of this study are firstly the fact that it is a large-scale birth cohort study consisting of approximately 100,000 pregnant women. To our knowledge, this is the only nationwide study that has verified the effect of physical activity on preterm delivery and mode of delivery in Japanese population. Second, as this was not an intervention study, physical activities other than exercise programs, such as work-related activities, housework, and leisure-time activities, could be taken into account. As such, all daily physical activities have been assessed quantitatively. Many papers on intervention studies report various program achievement rates (subjects with poor adherence are also present) and the fact that physical activities outside of the program are not considered; there is a drawback of exercise programs not being uniform in terms of type, intensity, and length of physical exercise. Those drawbacks were addressed in this study. Thirdly, multiple covariates (confounding factors) were identified and thus various influences could be considered and eliminated.

Our study has some limitations. First, the level of physical activity was assessed based on data collected via a self-administered questionnaire, and is therefore not an objective assessment. Second, the questions *per se* referred to a certain period of time during pregnancy and therefore did not necessarily cover the level of physical activity in the entire pregnancy period. Many subjects responded to the questionnaires during the second or third trimester of their pregnancy, so the status of physical activity in early pregnancy was not reflected. As such, there is recall bias in the level of pre-pregnancy physical activity. Third, even with incomplete responses to the IPAQ questionnaire that was used to calculate the amount of physical activity, METs were calculated based on the partial responses. As such, in the analysis of term of delivery and that of mode of delivery, the METs value obtained was calculated based on incomplete responses in 1478 and 1380 respondents, respectively. Therefore, it is likely that the influence of the level of physical activity was underestimated. Fourth, although factors that can affect the level of physical activity such as past history, health status, and pregnancy complications were considered when performing the analyses, the Very low and Low groups were assessed together with the “exercise not performed though it can be performed group” and “not in a state where exercise can be performed group”. We adjusted for threatened abortion, threatened preterm delivery, hypertension, and gestational hypertension as confounders, because these are causes of exercise restriction. However, we could not include placental previa, premature rupture of membrane, fetal growth restriction, and significant cardiac disease as confounders. As such, we could not completely eliminate the adverse influences extending to the “not in a state where exercise can be performed group”.

## Conclusion

In summary, pre-pregnancy physical activity had no negative effects on preterm birth and mode of delivery. In contrast, these may have been affected by exercise during pregnancy, as a low level of exercise appeared to increase the risk of preterm delivery and operative delivery (Caesarean and instrumental deliveries). Considering that pre-pregnancy physical exercise had no impact on pregnancy outcomes, this presents an opportunity to encourage women who normally do not habitually exercise to change their exercise habits even after becoming aware that they are pregnant.

## Supporting information

S1 Fig Participants inclusion flowchart with regard to the analysis of physical activities before pregnancy. (TIF)

S1 Table Amounts of physical activity before pregnancy (Mets・h/w). (XLSX)

S2 Table Maternal characteristics according to the term of delivery (n = 89,632) or the mode of delivery (n = 89,398). (XLSX)

S3 Table Association between physical activity level before pregnancy and preterm delivery. *p<0.05.
Adjusted for maternal age, current marital status, education, annual household income, alcohol intake during 2^nd^ 3^rd^ trimester, smoking status during 2^nd^ 3^rd^ trimester, exposure to tobbaco smoke during 2^nd^ 3^rd^ trimester, threatened abortion, threatened premature delivery, gestational diabetes, gestational hypertension, intrauterine infection, hypertension, diabetes, uterine fibroids, cervical cancer, previous preterm delivery, previous caesarean delivery, BMI before pregnancy, weight gain during pregnancy, SF-8 Physical Component Summary during pregnancy, total carorie intake during pregnancy, folic acid intake during pregnancy.
(XLSX)

S4 Table Association between physical activity level before pregnancy and mode of delivery. *p<0.05.
Adjusted for maternal age, current marital status, education, annual household income, alcohol intake during 2^nd^ 3^rd^ trimester, smoking status during 2^nd^ 3^rd^ trimester, exposure to tobbaco smoke during 2^nd^ 3^rd^ trimester, threatened abortion, threatened premature delivery, gestational diabetes, gestational hypertension, intrauterine infection, hypertension, diabetes, uterine fibroids, cervical cancer, previous caesarean delivery, BMI before pregnancy, weight gain during pregnancy, SF-8 Physical Component Summary during pregnancy, total carorie intake during pregnancy, folic acid intake during pregnancy.
(XLSX)
