# Supplementary figures and images for: Effects of physical activity during pregnancy on preterm delivery and mode of delivery: The Japan Environment and Children’s Study, birth cohort study
Source: PLoS One. 2018 Oct 29;13(10):e0206160. doi: 10.1371/journal.pone.0206160 (PMC6205641; doi:10.1371/journal.pone.0206160)

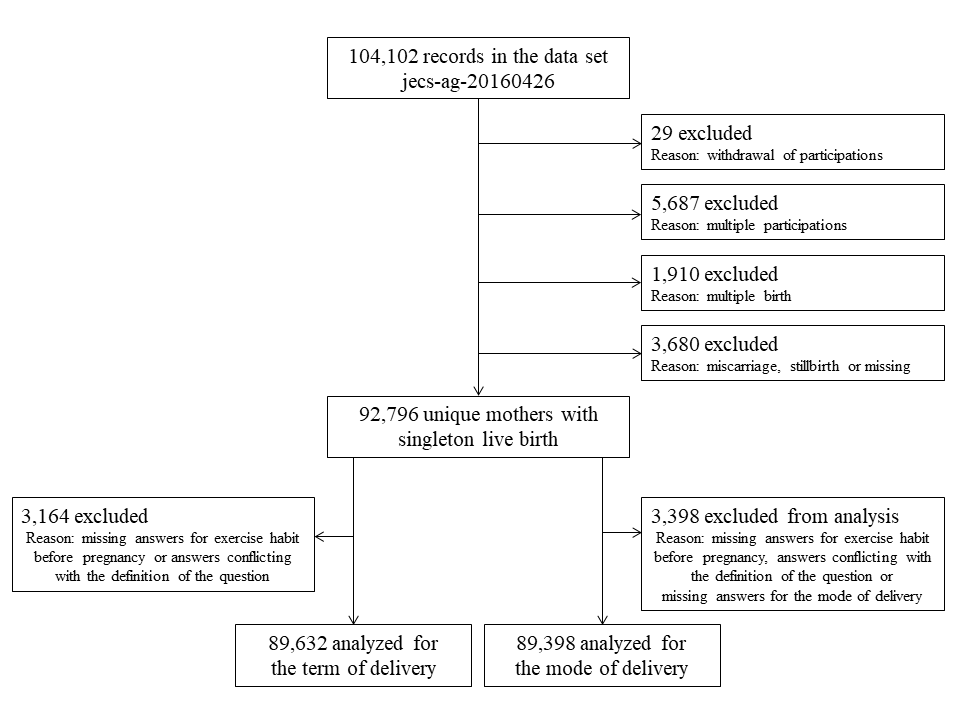

Supplement: S1 Fig — (TIF) [file pone.0206160.s001.tif]
